# Hierarchical multi-shell hollow micro–meso–macroporous silica for Cr(VI) adsorption

**DOI:** 10.1038/s41598-020-66540-6

**Published:** 2020-06-17

**Authors:** Roozbeh Soltani, Azam Marjani, Reza Soltani, Saeed Shirazian

**Affiliations:** 1 Department of Chemistry, Arak Branch, Islamic Azad University, Arak, Iran; 2 Department of Chemistry, Lorestan University, Khoramabad, Iran; 3 Department for Management of Science and Technology Development, Ton Duc Thang University, Ho Chi Minh City, Vietnam; 4 Faculty of Applied Sciences, Ton Duc Thang University, Ho Chi Minh City, Vietnam

**Keywords:** Nanoscale materials, Other nanotechnology, Chemistry, Analytical chemistry, Environmental chemistry, Inorganic chemistry, Materials chemistry

## Abstract

The development of easier, cheaper, and more effective synthetic strategies for hierarchical multimodal porous materials and multi-shell hollow spheres remains a challenging topic to utilize them as adsorbents in environmental applications. Here, the hierarchical architecture of multi-shell hollow micro–meso–macroporous silica with pollen-like morphology (MS-HMS-PL) has been successfully synthesized *via* a facile soft-templating approach and characterized for the first time. MS-HMS-PL sub-microspheres showed a trimodal hierarchical pore architecture with a high surface area of 414.5 m^2^ g^−1^, surpassing most of the previously reported multishelled hollow nanomaterials. Due to its facile preparation route and good physicochemical properties, MS-HMS-PL could be a potential candidate material in water purification, catalysis, and drug delivery. To investigate the applicability of MS-HMS-PL as an adsorbent, its adsorption performance for Cr(VI) in water was evaluated. Important adsorption factors affecting the adsorption capacity of adsorbent were systematically studied and Kinetics, isotherms, and thermodynamics parameters were computed *via* the non-linear fitting technique. The maximum capacity of adsorption computed from the Langmuir isotherm equation for Cr(VI) on MS-HMS-PL was 257.67 mg g^−1^ at 293 K and optimum conditions (pH 4.0, adsorbent dosage 5.0 mg, and contact time 90 min).

## Introduction

During the past decades, tremendous progress has been made in the design, synthesis, and application of hierarchical multi-shell hollow micro-meso-macroporous (MS-HM) architectures. These materials possess marvelous features, including low density, large surface area, the void spacing between the shells, high loading capacity, and good desorption performance, which endow them with widespread potential applications in water treatment, drug delivery, supercapacitors, fuel cells, nano-reactors, catalysis, dye-sensitized solar cells, and sensors^1–4^. These materials with triple or more hollow vesicles or lamellar shells are expected to yield better performances than their single- or double-shell nanoporous counterparts, although their synthesis routes are not straightforward similar to those with single- or double-shell owing to the increased complexity of the structure^4^.

The literature reveals that the nanoporous silica materials have attracted a great deal of attention compared with other porous materials due to their easily accessible starting materials, biodegradability, functionalizability, environmentally friendly nature, and high chemical and mechanical stability^5–9^. Hierarchically structured MS-HM silica (MS-HMS) materials have the advantages of both nanoporous silica and multi-shell hollow structures. Synthesis of MS-HMSs—mainly constituted of various sequences of interconnected pores of different sizes ranging from micro-(<2 nm), meso-(2–50 nm), to macropores (>50 nm)—offers an intelligent strategy to promote the adsorption properties, catalysis performance, and drug loading/releasing ability through minimizing diffusion barriers^6,10,11^. However, until now, there are relatively few studies that have reported the synthesis of MS-HMS materials. Zhao and co-workers (2010) reported a synthesis route for the preparation of carbon-silica composite with a multilayer vesicular structure using the aqueous emulsion-hydrothermal method from multiconstituent co-assembly of preformed resols, trimethyl benzene, Pluronic F127, and silicate oligomers in an acidic solution^12^. Yeh and co-workers (2011) reported the fabrication of triple-shelled silica nanospheres (with a surface area of 329.2 m^2^ g^−1^) through a hard-templating technique using polystyrene nanoparticles as a template^13^. A sacrificial template method, proposed by Ma and co-workers (2017), could also be used for the preparation of the triple-shelled hollow silica microspheres possessing a hierarchically porous structure with a surface area of 35 m^2^ g^−1^ ^14^.

Although these above-mentioned preparation methods report a multi-shell hollow structure, a tedious and complicated multi-step synthesis procedure or a low surface area silica product limited the availability or usability of the aforementioned templating methods. Another synthesis protocol for MS-HMS materials was introduced in 2019 by Soltani et al. where shell-in-shell functionalized silica hollow microspheres with mesoporous structure may be synthesized *via* a soft-templating method^15^. Although this method uses a simple and one-step synthesis procedure, however, this material has a low surface area of 29 m^2^ g^−1^. All the aforementioned MS-HMS materials have mesoporous layers or shells on their structures, and mesopores are the major constituent of these structures.

In this work, we report for the first time the synthesis of hierarchically architectured MS-HMS with pollen-like morphology (MS-HMS-PL) and uniform lamellar structure through a facile and one-step conventional soft-templating assisted hydrothermal method. Interestingly, MS-HMS displays a trimodal micro-meso-macrostructure. Hierarchically MS-HMS sub-microspheres with a majority of micropores in their lamellar shells and a high surface area of up to 414.5 m^2^ g^−1^ have not been synthesized until now. To investigate the adsorption efficiency and the applicability of the MS-HMS-PL for removal of toxic and highly mobile hexavalent chromium (Cr(VI)), its adsorption performance for Cr(VI) was evaluated. The adsorption technique, especially for removing toxic metals from water, has attracted a great deal of attention in recent years owing to its high efficiency, ease of operation, reusability, insensitivity to hazardous pollutants, use of a wide variety of adsorbents with designable structures, and availability of different adsorbents with relatively low-cost^5,6,15,16^. Among the potential toxic metals, Cr(VI) was chosen because it is generated mainly due to human anthropological activities (*e.g*., cement, leather tanning, electroplating, and textile industries) and is present in industrial wastes and possesses strong toxicity, mutagenicity, and carcinogenicity^16–18^. Important adsorption factors affecting the adsorption performance of the adsorbent toward Cr(VI) were systematically monitored and isotherms, kinetics, and thermodynamics parameters were estimated *via* the non-linear fitting method.

## Results and Discussion

### Synthesis of MS-HMS-PL

**Figure 1:**
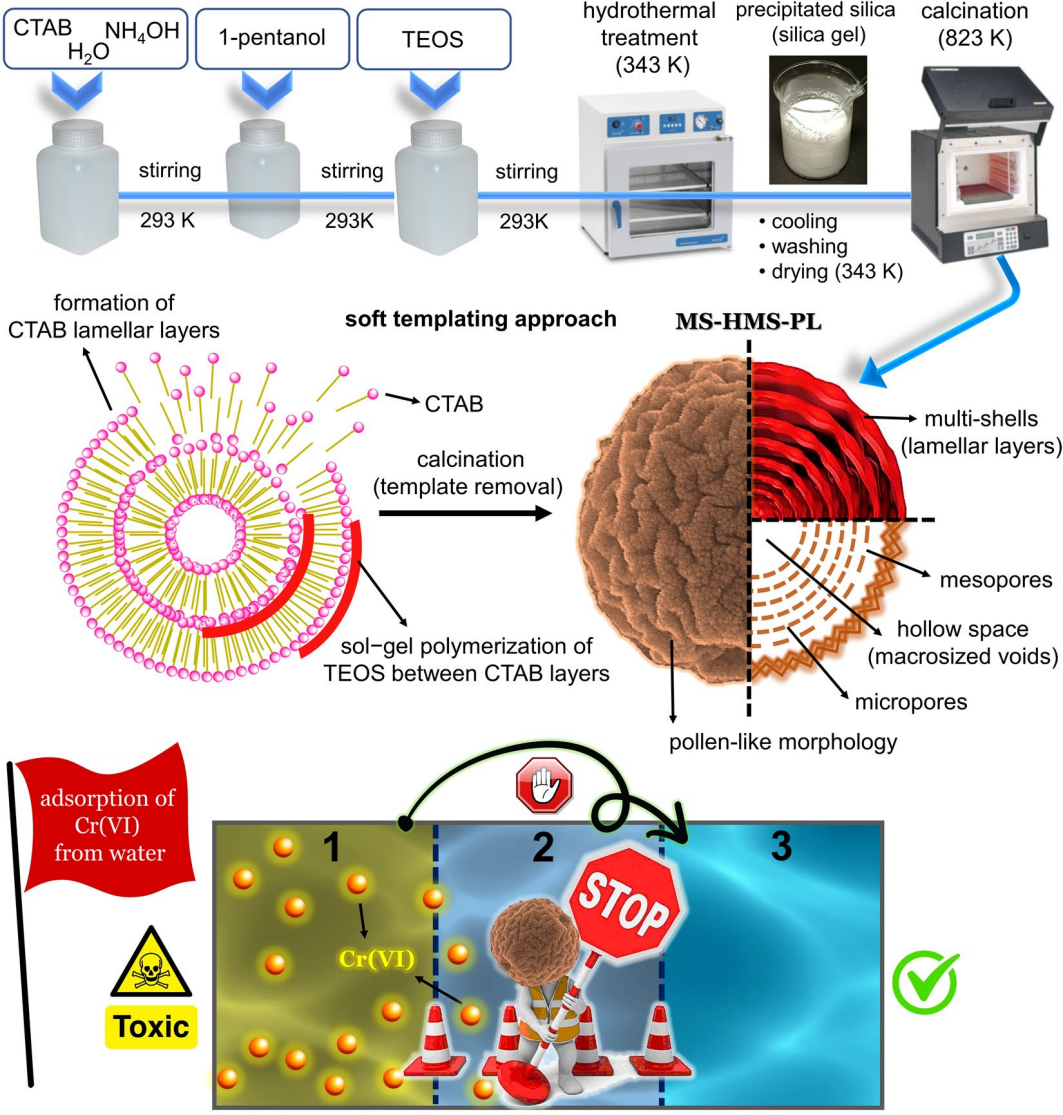
Schematic illustration of the synthesis procedure of hierarchically architectured MS-HMS-PL *via* soft-templating method and its application for Cr(VI) removal from the aqueous solution.

MS-HMS-PL was synthesized by a simple soft-templating hydrothermal method. The formation of multi-shell MS-HMS-PL implies the presence of multi-lamellar CTAB hollow spheres (Fig. 1, the formation of CTAB lamellar layers). The self-assembly of amphiphilic CTAB molecules in basic aqueous ammonia solution and the presence of 1-pentanol as co-surfactant leads to the formation of micelles and closed multi-layer hollow aggregates. The role of CTAB molecules is a structure-directing agent where *in-situ* polymerization of TEOS (in the basic condition) on the surface of preformed lamellar layers constructed by CTAB molecules leads to a hybrid organic-inorganic wall. After hydrothermal condition and complete polymerization of TEOS between CTAB layers (as depicted in Fig. 1), subsequent calcination was applied to remove the structure-directing agent and produce a stable MS-HMS-PL. In summary, we have developed a simple synthesis approach to the preparation of novel hierarchically architectured multi-shell hollow micro-meso-macroporous silica with pollen-like morphology. This material may have the potential for many uses in the fields of adsorption, analytical extraction, catalysis, carries, and drug release with prolonged-release time.

### Structure characterization of MS-HMS-PL

#### XRD analysis

**Figure 2:**
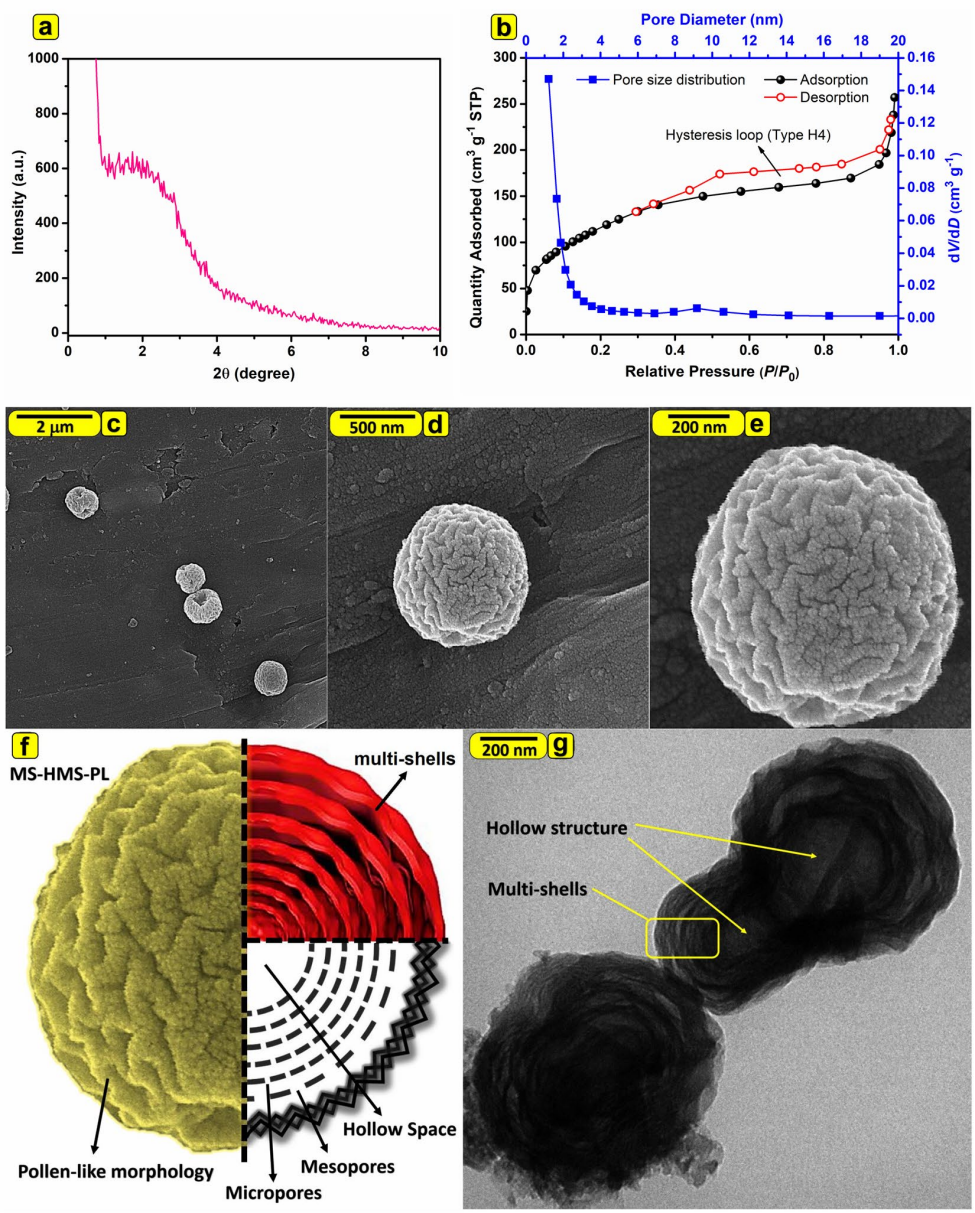
(**a**) L-XRD pattern of MS-HMS-PL. (**b**) N_2_ adsorption-desorption isotherms of MS-HMS-PL at 77 K and the BJH pore size distribution (pore diameter *vs* d*V*/d*P*) curve from the adsorption isotherm. (**c**–**e**) The FE-SEM, (**f**) the schematic structure representation, and (**g**) TEM micrograph of MS-HMS-PL.

To understand the crystalline structure of the MS-HMS-PL, L-XRD pattern of the sample is investigated. The L-XRD pattern of the MS-HMS-PL is shown in Fig. 2a. This silica material revealed only a broad diffraction peak at 2 *θ* 1° to 4°, implying the low ordering of silica framework in MS-HMS-PL.

#### FT-IR measurement

The FT-IR spectrum of MS-HMS-PL was recorded and is shown in Fig. S1. All characteristic absorption bands related to the nanoporous silica framework were observed for MS-HMS-PL (at 465 cm^−1^: bending mode of Si–O–Si; at 802 cm^−1^: symmetric mode of Si–O–Si vibration; at 964 cm^−1^: stretching mode of Si–OH; at 1088 and 1235 cm^−1^: asymmetric Si–O–Si vibration; 1626 cm^−1^: bending mode of surface adsorbed H_2_O; 3247 cm^−1^: O–H stretching modes of silanol groups) which were in close agreement with previously reported silica-based materials with nanoporous structures^9,19,20^. On a literature basis, a broad intense band between 3100-3750 cm^−1^ is attributed to the different silanol species (3650–3200 cm^−1^: O–H stretching modes of strongly hydrogen-bonded sialons in the silica framework; above 3650 cm^−1^: O–H stretching modes of isolated or free sialons)^21^.

#### Nitrogen adsorption-desorption isotherms

Nitrogen adsorption-desorption analysis was conducted with the aim of further investigating the structural feature of MS-HMS-PL. According to the IUPAC classification, MS-HMS-PL showed a combination of Type I(b) and IV isotherms with a Type H3 hysteresis loop at a relative pressure above 0.5 (Fig. 2b), suggesting the adsorption of N_2_ molecules in the hollow voids (macropores, Fig. 1)^22–24^. Type I(b) isotherms are representative of materials having pore size distributions in the range of wider micropores (<2 nm) and possibly narrow mesopores (<∼2.5 nm). Type IV isotherms are given by mesoporous materials. The BET surface area and total pore volume measured to be 414.5 m^2^ g^−1^ and 0.39 cm^3^ g^−1^ for MS-HMS-PL (Table S1). Using the Barrett–Joyner– Halenda (BJH) method, the average pore sized of MS-HMS-PL is estimated to be 1.22 nm. Accordingly, the macropores are related to the hollow space in the middle of the MS-HMS-PL sub-microspheres, and the micro-/mesopores are located within the multi-lamellar layers of MS-HMS-PL. The mesopores with the pore size in the range of 2-4 nm are also observed in the BJH pore size distribution curve. All of the above results clearly indicate that MS-HMS-PL sub-microspheres possess a trimodal hierarchical pore architecture including micropores (the major constituent of lamellar shells), mesopores (the minor constituent of lamellar shells), and macropores (in the middle of sub-microspheres). This trimodal hierarchical pore structure is given in a schematic representation in Fig. 1.

Some typical hollow single, double, and multi-shell nanomaterials reported in the last decade are summarized in Table 1, and their properties including their composition, synthesis method, surface area, and pore size characteristics are also given. The BET surface area of MS-HMS-PL was 414.5 m^2^ g^−1^, surpassing most of the previously reported hollow nanomaterials. Furthermore, as shown in Table 1, unlike the reported materials in which mesopores are the major constituent of structure, the major constituent of lamellar shells in MS-HMS-PL sub-microspheres is micropores. With its trimodal hierarchical architecture and relatively high surface area, MS-HMS-PL could be a potential candidate for adsorption, extraction, catalysis, and drug delivery applications.

**Table 1:** Selection of the textural properties of some hollow single, double, and multi-shell nanomaterials in the last decade (HT, ST, *S*_BET_, and *D* are the hard-templating method, soft-templating method, BET surface area, and pore size, respectively)^*a*^.

year	Material	Composition	method	*S*_BET_(m^2^g^−1^)	*D* (nm)	Ref.
2010	MLV-CS	Carbon-SiO_2_	HT	329	5.3	^12^
2010	MLV-S-Cal	SiO_2_	HT	598	14.4	^12^
2010	MLV-S-MWD	SiO_2_	HT	530	22.6	^12^
2010	MMSHNs-4	SiO_2_	ST	414	4.1	^23^
2010	MMSHNs-5	SiO_2_	ST	385	3.9	^23^
2010	MMSHNs-6	SiO_2_	ST	453	2.6	^23^
2011	single-shelled silica	SiO_2_	HT	155.2	<8	^13^
2011	double-shelled silica	SiO_2_	HT	243.9	~11	^13^
2011	triple-shelled silica	SiO_2_	HT	329.2	~11	^13^
2013	triple shells SnO_2_ HMSs	SnO_2_	HT	32.84	not reported	^35^
2013	quadruple shells SnO_2_ HMSs	SnO_2_	HT	36.27	not reported	^35^
2013	quintuple shells SnO_2_ HMSs	SnO_2_	HT	38.74	not reported	^35^
2014	double-shelled Mn_2_O_3_	Mn_2_O_3_	HT	27.71	not reported	^33^
2014	triple-shelled Mn_2_O_3_	Mn_2_O_3_	HT	36.55	not reported	^33^
2014	quadruple-shelled Mn_2_O_3_	Mn_2_O_3_	HT	30.22	not reported	^33^
2014	SS-TiO_2_-HNPs	TiO_2_	HT	63.6	not reported	^36^
2014	DS-TiO_2_-HNPs	TiO_2_	HT	128.4	not reported	^36^
2014	MS-TiO_2_-HNPs	TiO_2_	HT	171.3	4.1	^36^
2014	Co_3_O_4_ yolk–shell powders	Co_3_O_4_	HT	1.5-6.2	5-60	^37^
2015	Fe_3_O_4_@MnO_2_ BBHs	Fe_3_O_4_- MnO_2_	HT	247.9	3.7	^1^
2019	multi-shelled TAS-HMSs	SiO_2_	ST	29	<20	^15^
2020	MS-HMS-PL	SiO_2_	ST	414.5	1.22	This work

^*a*^MLV-CS: multilayer vesicle carbon–silica composite; MLV-S-Cal: MLV calcined silica; MLV-S-MWD: the silica vesicles from the microwave digestion method; MMSHNs: multi-shelled mesoporous silica hollow nanospheres; SS-TiO_2_-HNPs: single-shell TiO_2_ hollow nanoparticles; DS-TiO_2_-HNPs: double-shell TiO_2_-HNPs; MS-TiO_2_-HNPs: multi-shell TiO_2_-HNPs; TSHMs: triple-shell hollow microspheres; BBHs: ball-in-ball hollow spheres; TAS-HMSs: triamine-functionalized SiO_2_ hollow microspheres; MSHSs: multi-shelled hollow spheres.

#### FE-SEM and TEM micrographs

The FE-SEM micrographs of the MS-HMS-PL as shown in Fig. 2c–e revealed that this material is highly dispersed and possesses uniform pollen-like morphology. The magnified FE-SEM image (Fig. 2e) gave further information about the surface of the MS-HMS-PL with a wrinkled surface which can increase the accessible external surface area compared to the flat smooth surface. The TEM image of MS-HMS-PL (Fig. 2g) clearly shows that the particles possess a hollow spherical structure with a multi-lamellar shell. The schematic structure representation of MH-HMS-PL is shown in Fig. 2f.

### Application of MS-HMS-PL as an adsorbent in the removal of Cr(VI) from aqueous solution

The important factors including pH, the dosage of adsorbent, contact time, initial Cr(VI) concentrations, and temperature, affecting the adsorption performance of MS-HMS-PL were systematically monitored. The adsorption kinetics, isotherms, and thermodynamics of Cr(VI) on MS-HMS-PL in aqueous solutions were investigated by conducting a series of kinetics, isotherms, and thermodynamic studies. The experimental adsorption data were fitted to the various kinetics and isotherms by the nonlinear method. According to the literature, the nonlinear method is a better way to obtain the kinetics and isotherm parameters because the linearization of nonlinear kinetic and isotherm expressions usually distort the fit by changing the error distributions^5–7,25–27^. The nonlinear forms of different kinetic (Eqs. 5–8) and isotherm models (Eqs. 9–11), as well as thermodynamic equations (Eqs. 12–14), and their parameters are tabulated in Table 2.

**Table 2:** Non-linear form of Kinetic, isotherm, and thermodynamic equations and their parameters.

Eq.	Kinetic equations		
5	PFO	${Q}_{{\rm{t}}}={Q}_{{\rm{e}},{\rm{cal}}.}\cdot (1-{e}^{-{k}_{1}\cdot t})$	${Q}_{{\rm{e}},{\rm{cal}}.}$: the calculated adsorption capacity at any time *t* (mg g^−1^) *k*_1_: the PFO rate constant of adsorption (min^−1^)
6	PSO	${Q}_{{\rm{t}}}=\frac{{Q}_{{\rm{e}},{\rm{cal}}.}^{2}\cdot {k}_{2}\cdot t}{1+{Q}_{{\rm{e}},{\rm{cal}}.}\cdot {k}_{2}\cdot t}$	*k*_2_: the PSO rate constant of adsorption (g mg^−1^ min^−1^)$h(h={Q}_{{\rm{e}},{\rm{cal}}.}^{2}\cdot {k}_{2})$: the initial adsorption rate (mg g^−1^ min^−1^)
7	Elovich	${Q}_{t}=\frac{1}{\beta }\,\mathrm{ln}(\alpha \cdot \beta )\cdot t$	*α*: the initial adsorption rate (mg g^−1^ min^−1^)*β*: kinetic parameter related to the activation energy for chemisorption and extent of surface coverage (g mg^−1^)
8	IPD	${Q}_{{\rm{t}}}={k}_{{\rm{IPD}}}\cdot {t}^{0.5}+C$	*k*_IPD_: intra-particle diffusion rate constant (mg g^−1^ min^−0.5^)*C*: intra-particle diffusion constant related to the thickness of the boundary layer (mg g^−1^)
	**Isotherm equations**		
9	Langmuir	${Q}_{{\rm{e}}}=\frac{{Q}_{{\rm{m}},{\rm{cal}}.}{K}_{{\rm{L}}}{C}_{{\rm{e}}}}{1+{K}_{{\rm{L}}}{C}_{{\rm{e}}}}$	${Q}_{{\rm{m}},{\rm{cal}}.}$: the theoretical maximum adsorption capacity (mg g^−1^) *K*_L_: the Langmuir equilibrium constant (L mg^−1^) *R*_L_ (${R}_{{\rm{L}}}=[1/(1+{K}_{{\rm{L}}}\cdot {C}_{{\rm{e}}})]$): the separation factor (−)
10	Freundlich	${Q}_{{\rm{e}}}={K}_{{\rm{F}}}{C}_{{\rm{e}}}^{1/n}$	*K*_F_: Freundlich constant ((mg g^−1^) (L mg^−1^)^1/*n*^) *n*: adsorption intensity (−)
11	R-P	${Q}_{{\rm{e}}}=\frac{{K}_{{\rm{R}}-{\rm{P}}}{C}_{{\rm{e}}}}{1+{\alpha }_{{\rm{R}}-{\rm{P}}}{C}_{{\rm{e}}}^{{\rm{g}}}}$	*K*_R−P_: Redlich-Peterson isotherm constant (L g^−1^) *α*_R−P_: Redlich-Peterson isotherm constant (mg L^−1^)^−*g*^ *g*: Redlich-Peterson isotherm binding constant (0 < *g* < 1)
	**Thermodynamic equations**		
12	${K}_{{\rm{e}}}^{{\rm{o}}}=\frac{1000\cdot {K}_{L}\cdot MW\cdot {[adsorbate]}^{{\rm{o}}}}{\gamma }$		${K}_{{\rm{e}}}^{{\rm{o}}}$: thermodynamic equilibrium constant (−) *MW*: the molecular weight of the adsorbate (g mol^−1^) [*absorbate*]°: the unitary standard concentration of the adsorbate (1 mol L^−1^) *γ*: the coefficient of activity (−)
13	$\mathrm{ln}\,{K}_{{\rm{e}}}^{{\rm{o}}}=-\frac{\Delta {H}_{{\rm{ads}}.}^{{\rm{o}}}}{R}\cdot \frac{1}{T}+\frac{\Delta {S}_{{\rm{ads}}.}^{{\rm{o}}}}{T}$		*R*: the universal gas constant (J mol^−1^ k^−1^) $\Delta {H}_{{\rm{ads}}.}^{{\rm{o}}}$: standard enthalpy changes of adsorption (kJ mol^−1^) $\Delta {S}_{{\rm{ads}}.}^{{\rm{o}}}$: standard entropy changes of adsorption (J mol^−1^ K^−1^)
14	$\Delta {G}_{{\rm{ads}}.}^{{\rm{o}}}=\Delta {H}_{{\rm{ads}}.}^{{\rm{o}}}-T\cdot \Delta {S}_{{\rm{ads}}.}^{{\rm{o}}}$		$\Delta {G}_{{\rm{ads}}.}^{{\rm{o}}}$: standard Gibbs free changes of adsorption (kJ mol^−1^)

#### pH and adsorbent dosage

The influence of pH on the removal percentage of Cr(VI) at three different dosages of adsorbent was tested and is shown in Fig. S2. From Fig. S2, the maximum removal percentage achieved at pH 4.0 and exceeded ~93% at all three dosages of the adsorbent. At this solution pH, the removal percentages of ~93%, ~97%, and ~99% were obtained for adsorbent dosages of 2.0, 5.0, and 8.0 mg, respectively. Accordingly, pH 4.0 and adsorbent dosage of 5.0 mg were used for next adsorption studies as optimum conditions. The removal percentage decreased continuously with the increase of pH values and ~72% Cr(VI) was removed at pH 8.0 and 5.0 mg adsorbent dosage. Decreasing the solution pH below 4.0 leads to a decrease in the removal percentage and the adsorbent removed ~67% Cr(VI) at pH 2.0 and 5.0 mg adsorbent dosage. According to the literature, silica-based adsorbents are deprotonated above pH 4.5-5.0^10,28,29^ and this may be the reason for decreasing the removal percentage of Cr(VI) anions at higher pH as a consequence of electrostatic repulsion between Cr(VI) oxyanions and negatively charged surface of MS-HMS-PL. Also, the lower amount of removal percentage at lower pHs may be due to the high concentration of H_3_O^+^ cations in acidic media where the interactions of Cr(VI) anions with H_3_O^+^ cations are more effective than the adsorbent surface. At acidic aqueous media lower than 6.0, the dominant species are ${{\rm{HCrO}}}_{4}^{-}$ (pH 2.0-5.0) and ${{\rm{CrO}}}_{4}^{2-}$ (pH > 5.5).

#### Effect of time and initial concentration

**Figure 3:**
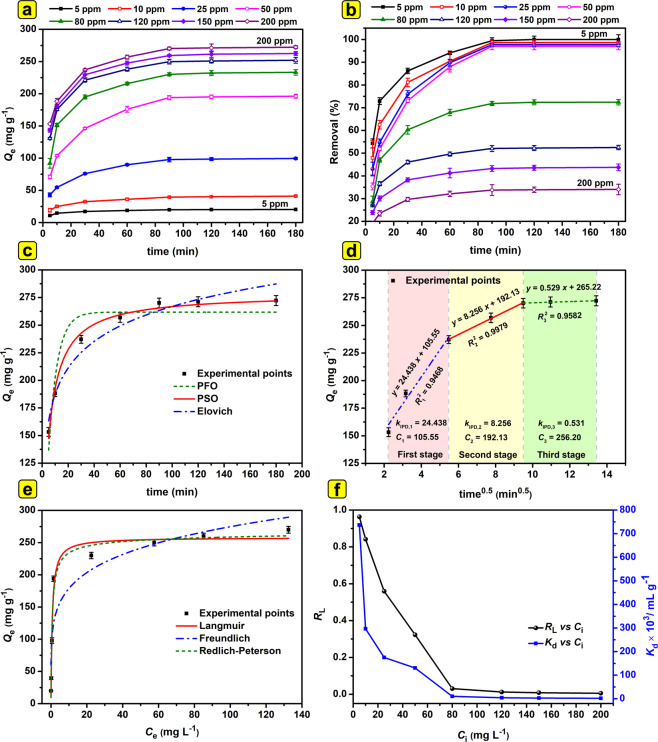
The effect of time and initial concentration on (**a**) adsorption capacity of adsorbent and (**b**) removal percentage of Cr(VI) under constant conditions (pH: 4.0; *W*: 5.0 mg; *V*: 20 ml; *C*_i_: 5–200 mg L^−1^; *T*: 293 K; shaking speed: 190 rpm; *t*: 5–180 min). (**c**) The experimental data and the nonlinear kinetics fitted to them and (**d**) the linear fitting of the IPD kinetic model (pH: 4.0; *W*: 5.0 mg; *V*: 20 ml; *C*_i_: 200 mg L^−1^; *T*: 293 K; *t*: 5–180 min). (**e**) The experimental data and the nonlinear isotherms fitted to them, and (**f**) the values of *R*_L_ and *K*_d_ *vs C*_i_ (pH: 4; *W*: 5.0 mg; *V*: 20 ml; *C*_i_: 5–200 mg L^−1^; *T*: 293 K; shaking speed: 190 rpm; *t*: 90 min).

The simultaneous influence of time and initial concentration changes on the adsorption capacity of adsorbent and removal percentage of Cr(VI) was monitored under constant conditions (Fig. 3a,b; figure caption for details). As shown in Fig. 3a, the adsorption capacity of the MS-HMS-PL increased with augmenting the initial concentration of Cr(VI) from 5 to 200 mg L^−1^. However, the removal percentage decreased with augmenting the initial concentration of Cr(VI) as shown in Fig. 3b. The experimental adsorption capacity after reaching equilibrium (*Q*_e,exp_/mg g^−1^) for certain initial concentrations are given in Table 3. When the initial concentration of Cr(VI) was 5 mg L^−1^, the adsorption capacity was 19.89 mg g^−1^ (%Removal = 99.46%). The adsorption capacity increased to 270.14 mg g^−1^ (%Removal = 33.77%) at an initial concentration of 200 mg L^−1^.

**Table 3:** Kinetic parameter values after non-linear fitting (pH = 4.0, *V* = 20 mL, *W* = 5.0 mg, *C*_i_ = 5–200 mg L^−1^, time = 5-180 min, *T* = 293 K, stirring rate = 190 rpm).

Model	Parameter	*C*_i_ (mg L^−1^/ ppm)							
		5	10	25	50	80	120	150	200
PFO	${Q}_{{\rm{e}},\exp }$ (mg g^−1^)	19.89	39.47	97.77	194.05	230.15	249.82	259.30	270.14
	${Q}_{{\rm{e}},{\rm{cal}}}$ (mg g^−1^)	19.24	37.75	93.83	187.36	224.32	243.28	252.56	261.72
	*k*_1_ (min^−1^)	0.1514	0.1168	0.090	0.0738	0.1038	0.1374	0.1430	0.1480
	*R*^2^	0.9077	0.8906	0.8920	0.9310	0.9542	0.9384	0.9108	0.8905
PSO	${Q}_{{\rm{e}},{\rm{cal}}}$ (mg g^−1^)	20.48	40.86	103.01	208.45	244.78	260.01	269.27	278.55
	*k*_2_ × 10^−4^ (g mg^−1^ min^−1^)	111.10	39.50	11.70	4.59	5.64	7.80	7.98	8.08
	*h* (mg g^−1^ min^−1^)	4.66	6.59	12.42	19.94	33.79	52.73	57.86	62.85
	*R*^2^	0.9872	0.9830	0.9773	0.9875	0.9866	0.9978	0.9946	0.9893
Elovich	*α* (mg g^−1^ min^−1^)	58.62	37.51	48.08	60.14	142.18	498.95	635.99	782.76
	*β* (g mg^−1^)	0.3944	0.1676	0.0598	0.0272	0.0260	0.0294	0.0292	0.0290
	*R*^2^	0.9301	0.9601	0.9652	0.9642	0.9159	0.9248	0.9418	0.9470
IPD	*k*_IPD,1_ (mg g^−1^ min^−0.5^)	1.810	3.93	9.975	22.334	29.308	26.306	25.512	24.438
	*C*_1_ (mg g^−1^)	7.66	11.304	21.75	25.94	39.89	80.42	92.07	105.55
	${R}_{1}^{2}$	0.7795	0.9405	0.9883	0.9431	0.7881	0.8909	0.9465	0.9468
	*k*_IPD,2_ (mg g^−1^ min^−0.5^)	0.665	1.744	5.466	11.985	8.778	7.173	7.448	8.256
	*C*_2_ (mg g^−1^)	13.62	22.83	46.27	81.35	147.19	182.06	189.10	192.13
	${R}_{2}^{2}$	0.9969	0.9967	0.9879	0.9917	0.9984	0.9981	0.9955	0.9979
	*k*_IPD,3_ (mg g^−1^ min^−0.5^)	0.106	0.412	0.417	0.540	0.746	0.530	0.772	0.529
	*C*_3_ (mg g^−1^)	18.91	35.58	93.89	189.03	223.45	244.90	252.35	265.22
	${R}_{3}^{2}$	0.9335	0.9986	0.9720	0.9636	0.9776	0.9582	0.7835	0.9582

The adsorption of Cr(VI) occurs in three consecutive steps:

(I) An initial fast step that lasts for 30 min
(II) The slower adsorption process in the range from 30 to 90 min
(III) An almost constant absorption step with very little variation in the adsorption rate where further increase in contact time does not reveal an increase in removal percentage of Cr(VI) or adsorption capacity of the adsorbent.

Consequently, 90 min was selected as optimum contact time to ascertain the equilibrium uptake of Cr(VI) to the MS-HMS-PL in the following experiments.

#### Kinetic and isotherm studies

In order to investigate the regulation of Cr(VI) uptake on MS-HMS-PL, four different kinetic models, *viz*., pseudo-first-order (PFO), pseudo-second-order (PSO), Elovich, and intra-particle diffusion (IPD), were used to fit the experimental data. Kinetics for the Cr(VI) adsorption onto MS-HMS-PL were systematically studied at eight different initial concentrations (5–200 mg L^−1^) and the results are given in Table 3. The corresponding curves in Fig. 3c are the PFO, PSO, and Elovich models after non-linear fitting at an initial concentration of 200 mg L^−1^ (figure caption for details). From Table 3, for non-linear kinetic models, the *R*^2^ values of the PSO kinetic model were higher than those of PFO and Elovich models at all the initial Cr(VI) concentrations. For the initial concentration of 200 mg L^−1^, Table 3, the *R*^2^ values of the PFO, PSO, and Elovich models were respectively 0.8905, 0.9893, 0.9470, implying that PSO kinetic model gives the best fit to the data. Also, the experimental adsorption capacity at equilibrium, *Q*_e,exp_ = 270.14 mg g^−1^, was very close to the calculated adoption capacity at equilibrium, *Q*_e,cal_ = 278.55 mg g^−1^, obtained from the PSO model at the initial concentration of 200 mg L^−1^. Accordingly, the adsorption process of Cr(V) by MS-HMS-PL could be well represented by the non-linear PSO kinetic model for all eight concentrations. Fitting of the nonlinear PFO, PSO, and Elovich kinetic models for eight different initial concentrations is depicted in Fig. S3.

IPD kinetic model (Eq. 8) was used so as to gain a better understanding of the mechanism involved in the Cr(VI) adsorption on the MS-HMS-PL. Figure 3d shows the three-stage-adsorption process in the plots of *Q*_t_ versus *t*^0.5^ for the adsorption of Cr(VI) on the adsorbent, implying that more than one mechanism affected the adsorption process. Adsorption of Cr(VI) by hierarchically architectured MS-HMS-PL occurs in the three consecutive stages according to Fig. 3d:

(I) The First stage is attributed to the fast-external surface adsorption throughout the boundary layer film (external mass transfer) of liquid surrounding the outside of MS-HMS-PL
(II) The second stage is fast external or internal adsorption at a site on the MS-HMS-PL surface and the adsorption energy will directly relate to the type of interaction between Cr(VI) and MS-HMS-PL
(III) The third stage refers to the intraparticle diffusion mechanism as a rate-controlling step (gradual absorption by diffusion of the Cr(VI) anions to MS-HMS-PL site either *via* a solid surface diffusion or *via* a pore diffusion mechanism through the liquid-filled pores).

Figure S4 is the plot of *Qt* vs. *t*^0.5^ at different initial Cr (VI) concentrations and corresponding IPD kinetic parameters are given in Table 3.

Parameters of adsorption isotherms for a target adsorbate are of great significance for evaluating and predicting the adsorption behavior of an adsorbent. Two two-parameter isotherm models (namely the Langmuir and Freundlich models; Table 1 Eqs. 9 and 10) and a three-parameter isotherm model (namely Redlich-Peterson model; eq. 11) were used to evaluate the equilibrium data for adsorptive removal of Cr(VI) anions by MS-HMS-PL. The isotherm curves for adsorption of Cr(VI) at 293 K are shown in Fig. 3e (figure caption for details) and corresponding isotherm parameters are tabulated in Table 4. For Cr(VI) adsorption on MS-HMS-PL, the Langmuir model provide the better approximation of *R*^2^ (*R*^2^ = 0.9784) than those of Freundlich (*R*^2^ = 0.8588) and R-P models (*R*^2^ = 0.9752). Moreover, the maximum adsorption capacity computed from the Langmuir model (*Q*_m,cal_ = 270.14 mg g^−1^) was close to the maximum adsorption capacity achieved in this experiment (*Q*_m,exp_ = 257.67 mg g^−1^) at 293 K, further demonstrating that the Langmuir isotherm model is better to express adsorption isotherm for Cr(VI) onto MS-HMS-PL.

**Table 4:** Isotherm parameter values after non-linear fitting (pH = 4.0, *V* = 20 mL, *W* = 5.0 mg, *C*_i_ = 5-200 mg L^−1^, time = 90 min, *T* = 293-323 K, stirring rate = 190 rpm).

Model	Parameter	*T* (K)			
		293	303	313	323
Langmuir	${Q}_{{\rm{m}},\exp }$ (mg g^−1^)	270.14	282.89	291.51	302.02
	${Q}_{{\rm{m}},{\rm{cal}}}$ (mg^−1^)	257.67	267.99	275.55	281.18
	*K*_L_ (L mg^−1^)	1.412	1.572	1.751	1.853
	*R*^2^	0.9784	0.9694	0.9780	0.9740
Freundlich	*K*_F_ ((mg g^−1^)(L mg^−1^)^1/*n*^)	116.16	116.48	117.92	124.27
	*n* (−)	5.37	5.08	4.97	5.11
	*R*^2^	0.8588	0.8430	0.8665	0.8632
R-P	*K*_R−P_(L g^−1^)	401.36	407.92	434.07	579.82
	*α*_R−P_ (mg L^−1^) ^−*g*^	1.691	1.651	1.794	2.397
	g (−)	0.9802	0.9803	0.9678	0.9625
	*R*^2^	0.9752	0.9646	0.9771	0.9734

Figure S5 discloses non-linear isotherm plots of Cr(VI) adsorption on the MS-HMS-PL at four different temperatures and their corresponding isotherm parameters are given in Table 4. The values of *Q*_m,cal_ calculated for the Langmuir model for adsorption of Cr(VI) onto MS-HMS-PL were estimated to be 257.67 (*Q*_m,exp_ =270.14 mg g^−1^), 267.99 (*Q*_m,exp_ = 282.89 mg g^−1^), 275.55 (*Q*_e,exp_ = 291.51 mg g^−1^), and 281, 18 mg g^−1^ (*Q*_m,exp_ = 302.02 mg g^−1^) at 293, 303, 313, and 323 K, respectively.

A useful dimensionless parameter called separation factor (*R*_L_, Table 2) is introduced in Langmuir’s model to suggest the type of adsorption process where the values of *R*_L_ = 0, *R*_L_ = 1, *R*_L_ > 1, and 0 <*R*_L_ < 1 imply that the adsorption process is irreversible, linear, unfavorable, and favorable, receptivity^8,24^. The values of *R*_L_ for adsorption of Cr(VI) onto MS-HMS-PL, as shown in Fig. 3f, lie between zero and unity, indicative of a favorable adsorption process.

Distribution coefficient values (*K*_d_, mL g^−1^, Eq. 4) could also be used to gain more insight into the favorability of the adsorption process under set conditions: *K*_d_∼10^2^ (∼500 mL g^−1^): acceptable; *K*_d_∼10^3^ (∼5000 mL g^−1^): very good; *K*_d_ ≥ 10^4^ (∼50000 mL g^−1^): outstanding^7,15,30,31^. In a removal process, the larger *K*_d_ values, the more affective the solid adsorbent is at holding and capturing the adsorbate. The *K*_d_ values for adsorption of Cr(VI) on MS-HMS-PL (Fig. 3f) were in the range of 2.04×10^3^–7.37×10^5^ mL g^−1^ in different initial Cr(VI) concentrations (5–200 mg L^−1^). At initial concentrations of 5-50 mg L^−1^ *K*_d_ values ~10^5^ were obtained. *K*_d_ values ~10^4^ were obtained at initial concentration of 80 mg L^−1^, and *K*_d_ values ~10^3^ were attained between 120-200 mg L^−1^ initial concentration. Consequently, from the experimentally obtained values of *K*_d_, MS-HMS-PL possesses outstanding (for *C*_i_ = 5–50 mg L^−1^), very good (for *C*_i_ = 80 mg L^−1^), and acceptable (for *C*_i_ = 120–200 mg L^−1^) adsorption performance for removal of Cr(VI) from aqueous solution depending on the initial Cr(VI) concentration at constant conditions (pH: 4; *W*: 5.0 mg; *V*: 20 ml; *C*_i_: 5–200 mg L^−1^; *T*: 293 K; shaking speed: 190 rpm; *t*: 90 min).

### Effect of temperature and thermodynamic studies

**Figure 4:**
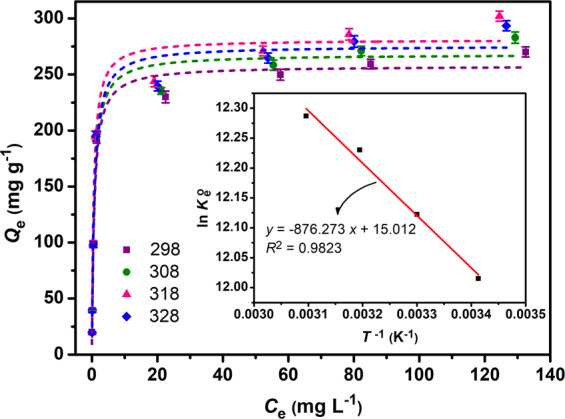
The effect of temperature on the adsorption capacity of MS-HMS-PL toward Cr(VI) from aqueous solution and its corresponding thermodynamic curve (inset) at 293, 303, 313, and 323 K under constant conditions (pH: 4.0; *W*: 5.0 mg; *V*: 20 ml; *C*_i_: 200 mg L^−1^; shaking speed: 190 rpm; *t*: 90 min).

The influence of solution temperature on the adsorption capacity of adsorbent for Cr(VI) is shown in Fig. 4. The adsorption capacity increased as the solution temperature increased (Table 5, Fig. 4). To systematically characterize the physicochemical behavior of Cr(V) removal and to get a better insight into the mechanism of adsorption, the thermodynamic parameters of adsorption, *viz*., $\Delta {G}_{{\rm{ads}}.}^{{\rm{o}}}$, $\Delta {H}_{{\rm{ads}}.}^{{\rm{o}}}$, and $\Delta {S}_{{\rm{ads}}.}^{{\rm{o}}}$, were estimated. These crucial parameters could be estimated from the temperature-dependent isotherms using the Eqs. (12–14) given in Table 1. Soltani *et al*.^5,8,24^ and Lima *et al*.^32^ reported that the correct approach to calculating the aforementioned constants for an adsorption system is to perform non-linear fitting of the isotherms (usually Langmuir model) of the adsorption at various temperatures. For these calculations, it is considered that the aqueous solution of Cr(VI) is very diluted to consider that *γ* (the coefficient of activity, Eq. 12) is unitary. Thermodynamic equilibrium constants (${K}_{{\rm{e}}}^{{\rm{o}}}$, Eq. 12) for each temperature could be calculated from the corresponding *K*_L_ values for each isotherm after fitting the Langmuir equation to the adsorption isotherms (Eq. 9). The values of $\Delta {H}_{{\rm{ads}}}^{{\rm{o}}}$ and $\Delta {S}_{{\rm{ads}}}^{{\rm{o}}}$ can be estimated from the slope and intercept of van’t Hoff equation (the plot of In ${K}_{{\rm{e}}}^{{\rm{o}}}$ versus 1/*T* in Eq. 13), respectively. The calculated thermodynamic parameters are given in Table 5. According to the literature, the $\Delta {G}_{{\rm{ads}}.}^{{\rm{o}}}$ (kJ mol^−1^) and $\Delta {H}_{{\rm{ads}}}^{{\rm{o}}}$ (kJ mol^−1^) values can explain the type of interaction using the following guideline: $\,\Delta {H}_{{\rm{ads}}}^{{\rm{o}}}$ > 60, chemical bonding forces, $\Delta {H}_{{\rm{ads}}}^{{\rm{o}}}$ ≈ 40, exchange of dentate; 4 <$\Delta {H}_{{\rm{ads}}}^{{\rm{o}}}$ <10, van der Waals interactions; 2 < $\Delta {H}_{{\rm{ads}}}^{{\rm{o}}}$ < 40, hydrogen bonding forces; 2 < $\Delta {H}_{{\rm{ads}}.}^{{\rm{o}}}$ < 29, dipole-dipole interactions; −20 < $\Delta {G}_{{\rm{ads}}}^{{\rm{o}}}$ < 0, physisorption; −400 < $\Delta {G}_{{\rm{ads}}}^{{\rm{o}}}$ < −80, chemisorption^10,24,33,34^. For the adsorption of Cr(VI), the $\Delta {G}_{{\rm{ads}}}^{{\rm{o}}}$ value ranged from −33 <$\Delta {G}_{{\rm{ads}}}^{{\rm{o}}}$ < −29, indicating that the adsorption process of Cr(VI) on MS-HMS-PL was more of a spontaneous physisorption phenomenon rather than that of chemisorption. $\Delta {H}_{{\rm{ads}}}^{{\rm{o}}}$ (+7.29 kJ mol^−1^) value, at the investigated temperatures, reveals that the adsorption process is the result of physical interactions such as electrostatic forces and van der Waals forces. Furthermore, the positive value of $\Delta {H}_{{\rm{ads}}}^{{\rm{o}}}$ clearly signifies that the interaction between the adsorbent and Cr(VI) anions is endothermic which was confirmed by the increase in adsorption capacity with increasing temperature. The positive value of $\Delta {S}_{{\rm{ads}}}^{{\rm{o}}}$ (+0.13 kJ mol^−1^ K^−1^) means the randomness enhancement at the Cr(VI) solution/MS-HMS-PL interface in the adsorption process.

**Table 5:** Thermodynamic parameters for adsorption of Cr(VI) on MS-HMS-PL (pH = 4.0, *V* = 20 mL, *W* = 5.0 mg, *C*_i_ = 5-200 mg L^−1^, stirring rate = 190 rpm).

$\Delta {{\boldsymbol{G}}}_{{\bf{a}}{\bf{d}}{\bf{s}}.}^{{\bf{o}}}$ kJ mol^−1^					
293 K	303 K	313 K	323 K	$\Delta {{\boldsymbol{H}}}_{{\bf{a}}{\bf{d}}{\bf{s}}.}^{{\bf{o}}}$ kJ mol^−1^	$\Delta {{\boldsymbol{S}}}_{{\bf{a}}{\bf{d}}{\bf{s}}.}^{{\bf{o}}}$ kJ mol^−1^ K^−1^
−29.28	−30.53	−31.78	−33.03	+7.29	+0.13

### Comparative study

A comparison of the optimal conditions and adsorption performance of MS-HMS-PL for the adsorption of Cr(VI) with those of other adsorbent is shown in Table 6. It is clear that MS-HMS-PL showed high adsorption capacity for Cr(VI), calculated from Langmuir fitting, as compared to different synthetic, natural, and modified adsorbents. Such an outstanding removal capacity of MS-HMS-PL for Cr(VI) may be directly attributed to its high surface area and trimodal micro-meso-macroporous architecture which may provide fast mass transfer for adsorbate.

**Table 6:** The comparison adsorption capacity of Cr(VI) under optimal conditions obtained by various adsorbents^*a*^.

Adsorbent	*Q*_m,cal_ (mg g^−1^)	pH	T (K)	*t* (min or h)	Ref.
bismuth hollow nanospheres	17.5	2.0	RT	—	^38^
Fe_3_O_4_/GO	32.33	4.5	293	5 h	^39^
EMCMCR	51.81	2.0	293	6–10 min	^40^
MSP	53.60	2.0	298	120 min	^41^
chitosan flakes	102	3.0	293	—	^42^
NMA-LDOs	103.4	—	303	150 min	^43^
magnetic poly(GMA–EGDMA) beads	140.6	2.0	298	120	^44^
G–MgAl-LDH nanocomposite	172.55	2.0	293	24 h	^45^
PANI@NC nanocomposites	198.04	1.0	298	480 min	^46^
MCS	200	natural	303	60	^47^
MHCSs	200	3.0	298	∼700 min	^48^
MI-Cl-KCC-1	428	3.0–4.0	298	40 min	^5^
MS-HMS-PL	257.67	4.0	293	90 min	this study

^*a*^Fe3O4/GO: porous Fe_3_O_4_ hollow microspheres/graphene oxide composite; EMCMCR: ethylenediamine-modified cross-linked magnetic chitosan resin; MSP: surfactant–modified serpentine; NMA-LDHs: Ni/Mg/Al layered double hydroxides; GMA–EGDMA: Glycidyl methacrylate-ethyleneglycol dimethacrylate; G–MgAl-LDOs: Graphene/MgAl-layered double oxides; PANI@NC: polyaniline grown on N-doped carbon nanoparticles; MCS: modified corn stalks; MHCSs: magnetic hollow carbon nanospheres; MI-Cl-KCC-1: N-methylimidazolium-functionalized KCC-1.

## Conclusion

In this study, a hierarchical multi-shell hollow micro-meso-macroporous silica with pollen like morphology designated as MS-HMS-PL was synthesized *via* a facile soft-templating method. BET and TEM analyses revealed that MS-HMS-PL sub-microspheres possess a trimodal hierarchical pore structure including micropores (the major constituent of lamellar shells), mesopores (the minor constituent of lamellar shells), and macropores (in the middle of sub-microspheres). With its high surface area (414.5 m^2^ g^−1^), multi-shell structure, and facile synthesis route, MS-HMS-PL cloud be a good candidate for some applications such as adsorption, extraction, catalysis, and drug delivery. Accordingly, the adsorption performance of MS-HMS-PL for removal of Cr(VI) from aqueous solution was systematically studied. The effect of important adsorption factors—*viz*., pH. adsorbent dosage, contact time, initial Cr(VI) concentration, and temperature—on the adsorption performance of MS-HMS-PL for Cr(VI) removal was evaluated. Kinetics, isotherms, and thermodynamic investigations were conducted to determine the adsorption mechanism and important parameters of adsorbent, and the non-linear fitting method was applied to calculated the kinetics and isotherms parameters. The estimated maximum adsorption capacity, according to the Langmuir model, was found to be 257.67 mg g^−1^ at pH 4.0, solution volume of 40 mL, the adsorbent dosage of 5.0 mg, the contact time of 90 min, stirring rate of 190 rpm, and solution temperature of 293 K.

## Methods

### Materials

Cetrimonium bromide (CTAB, ≥98.0%) and potassium dichromate (K_2_Cr_2_O_7_, ≥99.5%) were purchased from Sigma-Aldrich (Buchs, Switzerland). 1-pentanol (≥98.5%), ammonium hydroxide (NH_4_^+^OH^−^, 25% in water), silicon tetraethoxide (TEOS, ≥99.0%), sodium hydroxide pellets (NaOH, 99%), and fuming hydrochloric acid (HCl, 37%) were purchased from Merck (Darmstadt, Germany). Ethanol (96.0%) was prepared from Bidestan Co. (Ghazvin, Iran). Deionized (DI) water was utilized for the adsorption investigations and preparation of the adsorbent.

### Synthesis of MS-HMS-PL

In a typical synthesis route, in a 250 mL bottle made from high-density polypropylene (HDPP) with a leakproof screw cap, 1.800 g CTAB was dissolved in 90 mL of pure water with magnetically stirring at 293 K for 10 min. Then, 7.7 mL of NH_4_^+^OH^−^ was added to the bottle and the mixture was stirred for 20 min. Afterward, 2.7 mL of 1-pentanol was added to the mixture and stirred for 15 min followed by adding 7.5 mL of TEOS. The mixture was stirred at 293 K for 2 h and then maintained at an electric oven (343 K) for 24 h. The obtained white gel was washed with pure water and ethanol (96%) and dried at 333 K for 24 h. Finally, the white powder was calcined at 823 K for 6 h so as to remove the organic template CTAB.

### Batch experiments

Batch adsorption studies were conducted in 50-mL HDPP bottles with a leakproof screw cap and a constant working volume of 20 mL in an incubator-shaker apparatus at a shaking speed of 190 rpm. 1000 mg L^−1^ stock solution of Cr(VI) was prepared by mixing the appropriate mass of K_2_Cr_2_O_7_ in pure water. Working standard solutions with desired Cr(VI) concentrations (5-200 mg L^−1^) were prepared by suitable dilutions of Cr(VI) stock solution just before use. For each experiment, a certain amount of adsorbent (2.0, 5.0, 8.0, and 15.0 mg) was transferred to a 50-mL HDPP bottle containing 20 mL of Cr(VI) solutions with desired pH (2.0–8.0) and Cr(VI) concentration (5–200 mg L^−1^), and then agitated at 190 rpm at a certain temperature (293, 303, 313, and 323 K). The pH of the solutions was adjusted with 0.1 mg L^−1^ NaOH and HCL solutions. After specific time intervals (5–200 min), the Cr(VI)-loaded adsorbent particles were separated by centrifugation (3500 rpm, 5 min). The adsorption capacities of adsorbent at any time *t* (*Q*_t_, mg g^−1^), the adsorption capacity at equilibrium (*Q*_e_, mg g^−1^), and removal percentage were determined according to the following formula:

1 $${Q}_{{\rm{t}}}=({C}_{{\rm{i}}}-{C}_{{\rm{t}}})\cdot V/W$$

2 $${Q}_{{\rm{e}}}=({C}_{{\rm{i}}}-{C}_{{\rm{e}}})\cdot V/W$$

3 $$ \% \,{\rm{Removal}}=100\cdot ({C}_{{\rm{i}}}-{C}_{{\rm{e}}})/{C}_{{\rm{i}}}$$

where *C*_i_ is the initial adsorbate concentration (mg L^−1^), *C*_t_ is the adsorbate concentration at a certain time *t* (mg L^−1^), *C*_e_ is the adsorbate concentration at equilibrium (mg L^−1^), *V* is the volume of the liquid phase (L), and *W* is the mass of the adsorbent (mg).

The values of distribution coefficient (*K*_d_, mL g^−1^) at equilibrium can be calculated from the following formula:

4 $${K}_{{\rm{d}}}=\frac{({C}_{{\rm{i}}}-{C}_{{\rm{e}}})\cdot V}{{C}_{{\rm{i}}}\cdot W}=\frac{{Q}_{{\rm{e}}}}{{C}_{{\rm{e}}}}$$

### Characterization

Low angle X-ray diffraction (L-XRD) analysis, Fourier transform infrared (FT-IR) spectroscopy, nitrogen adsorption-desorption measurement at 77 K, field emission scanning electron microscopy (FE-SEM), and transmission electron microscopy (TEM), were used for the characterization of the sample. Also, flame atomic absorption spectroscopy (FAAS) was used for the determination of the Cr(VI) concentration in the aqueous solutions. The details of the apparatus used in this work are given in Supplementary Information (S1. Experimental section, Apparatus).

## Supplementary information

Supplementary information.
