# Supplementary material for: Hierarchical multi-shell hollow micro–meso–macroporous silica for Cr(VI) adsorption
Source: Sci Rep. 2020 Jun 17;10:9788. doi: 10.1038/s41598-020-66540-6 (PMC7300025; doi:10.1038/s41598-020-66540-6)
Supplement: Supplementary file 1 — Supplementary information. [file 41598_2020_66540_MOESM1_ESM.docx]

**Supplementary Information**

Hierarchical multi-shell hollow micro–meso–macroporous silica for Cr(VI) adsorption

**Roozbeh Soltani^a^, Azam Marjani^a^, Reza Soltani^b^, Saeed Shirazian^c,d^***

^a^ *Department of Chemistry, Arak Branch, Islamic Azad University, Arak, Iran*

^b^ *Department of Chemistry, Lorestan University, Khoramabad, Iran*

^c^ *Department for Management of Science and Technology Development, Ton Duc Thang University, Ho Chi Minh City, Vietnam*

^d^ *Faculty of Applied Sciences, Ton Duc Thang University, Ho Chi Minh City, Vietnam*

*Corresponding author: Saeed Shirazian; E-mail: [saeed.shirazian@tdtu.edu.vn](mailto:saeed.shirazian@tdtu.edu.vn)

**S1. Experimental section**

**Apparatus.** The structural properties of the materials were examined by low angle X-ray diffraction (L-XRD, Philips X’pert MPD diffractometer, Eindhoven, Netherland) technique with Cu *K*a radiation (*λ*=0.1542 nm) at 45 kV and 100 mA.

Fourier transform infrared (FT-IR, Avatar 370, Thermo Nicolet, USA) spectra of the samples were recorded from 4000 to 400 cm^–1^ region. Dried KBr was mixed with the powder samples and pressed as pallets for the measurements.

To measure the porosity and adsorption behavior of the samples, the Brunauer-Emmett-Teller (BET) method was used, employing a volumetric N_2_ adsorption-desorption (at 77K) apparatus, BELSORP-mini II (BEL Japan Inc., Osaka, Japan).

Field emission scanning electron microscope (FE-SEM, MIRA3 TESCAN-XMU, Kohoutovice, Czech Republic) equipped with energy dispersive spectrometer (EDS) was applied for observation of the morphology of the samples.

Transmission electron microscopy (TEM, Philips CM120 microscope, Eindhoven, Netherland) images were recorded with an accelerator voltage of 100 kV.

The concentrations of hexavalent chromium in the aqueous solution before and after adsorption procedure were measured using flame atomic absorption spectroscopy (FAAS, Perkin-Elmer Model A300 (Norwalk, CT, USA)).

The experimental adsorption data were fitted to the various kinetics and isotherms by the non-linear method. using the statistical analysis function in Origin Pro 8.6 software (Origin Lab Corporation, Northampton, USA).

**Table S1.** Structural characteristics of the MS-HMS-PL (TPV: total pore volume; *D*_BJH_: average pore diameter according to the BJH method). These data are calculated by using the nitrogen adsorption–desorption isotherms.

| Surface area (m^2^ g^–1^) | | |  |  |  |
| --- | --- | --- | --- | --- | --- |
| BET | Langmuir | BJH |  | TPV (cm^3^ g^–1^) | *D*_BJH_ (nm) |
| 414.5 | 462.1 | 218.8 |  | 0.39 | 1.2 |


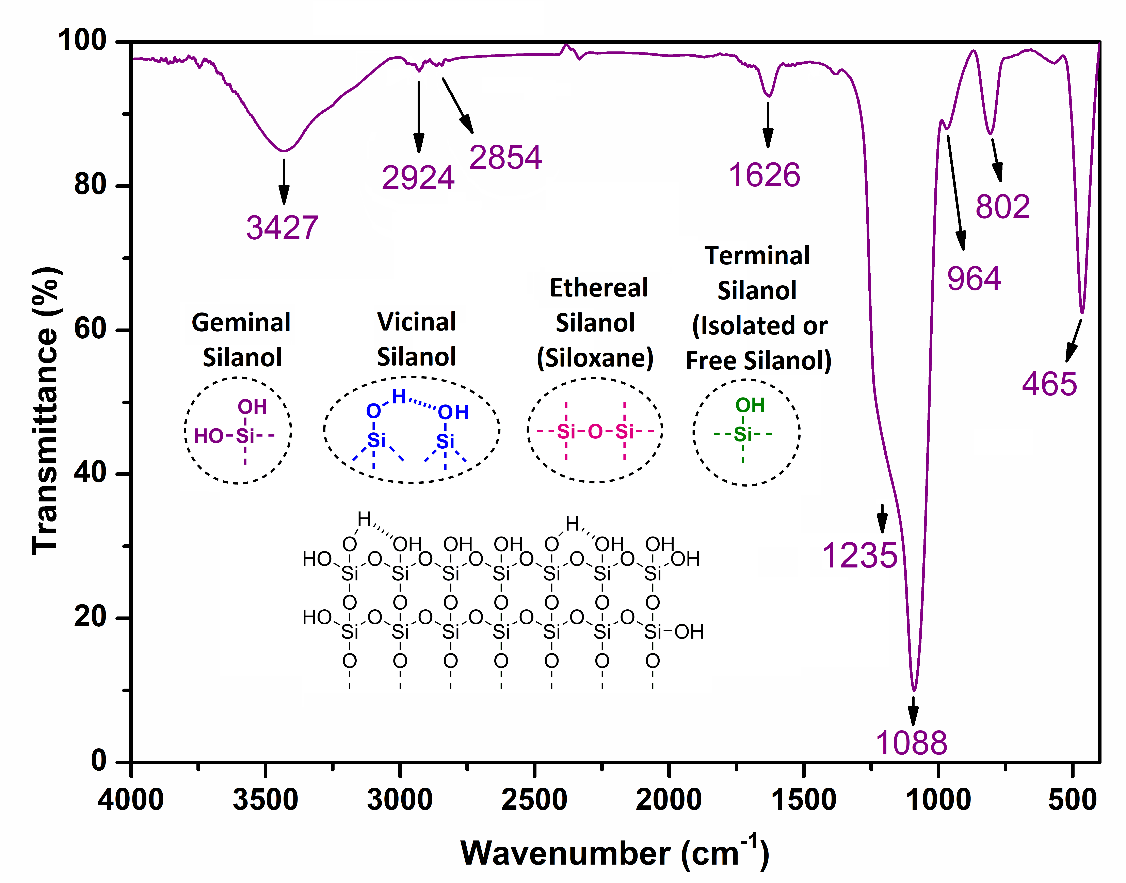


**Figure S1.** FT-IR spectrum of MS-HMS-PL.


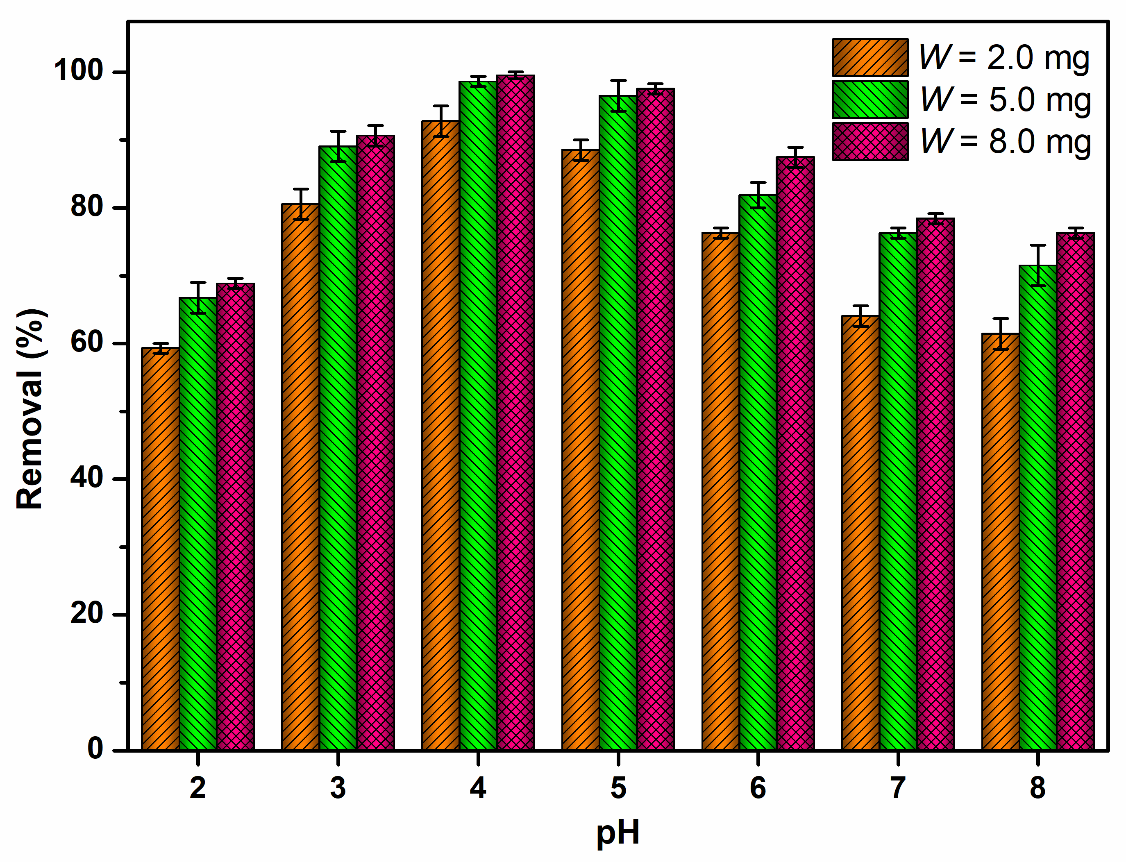


**Figure S2.** The effect of adsorbent dosage and solution pH on removal percentage of Cr(VI) by MS-HMS-PL under constant conditions (pH: 2.0-8.0; $W$: 2.0-8.0 mg; $V$: 20 ml; $C_{i}$: 50 mg L^–1^; $T$: 293 K; shaking speed: 190 rpm; $t$: 180 min).


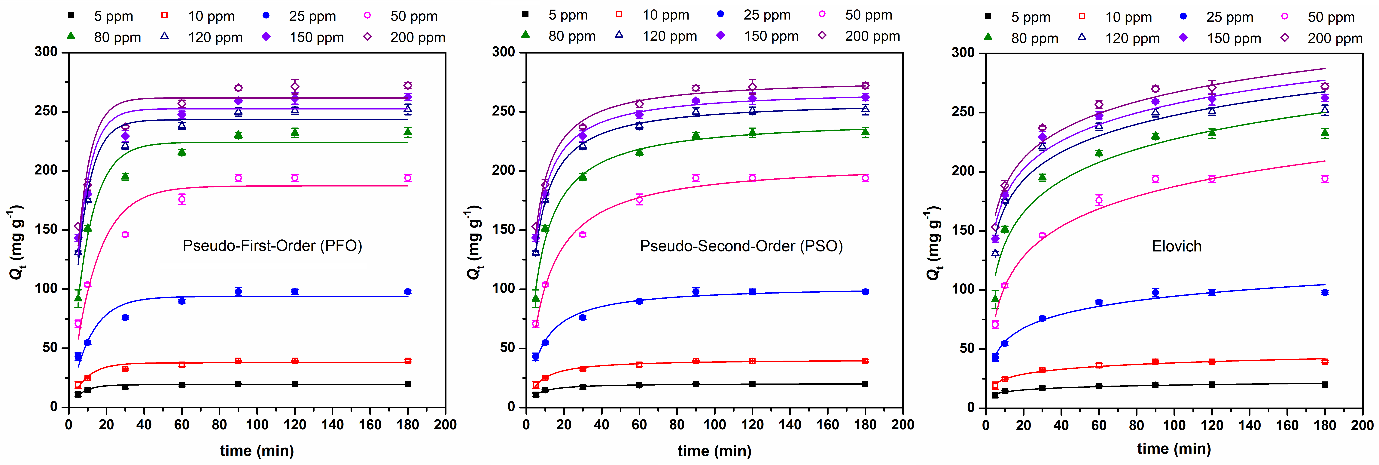


**Figure 3.** The experimental data and the nonlinear kinetics fitted to them for different initial concentrations (pH: 4.0; $W$: 5.0 mg; $V$: 20 ml; $C_{i}$: 5-200 mg L^–1^; $T$: 293 K; $t$: 5-180 min).


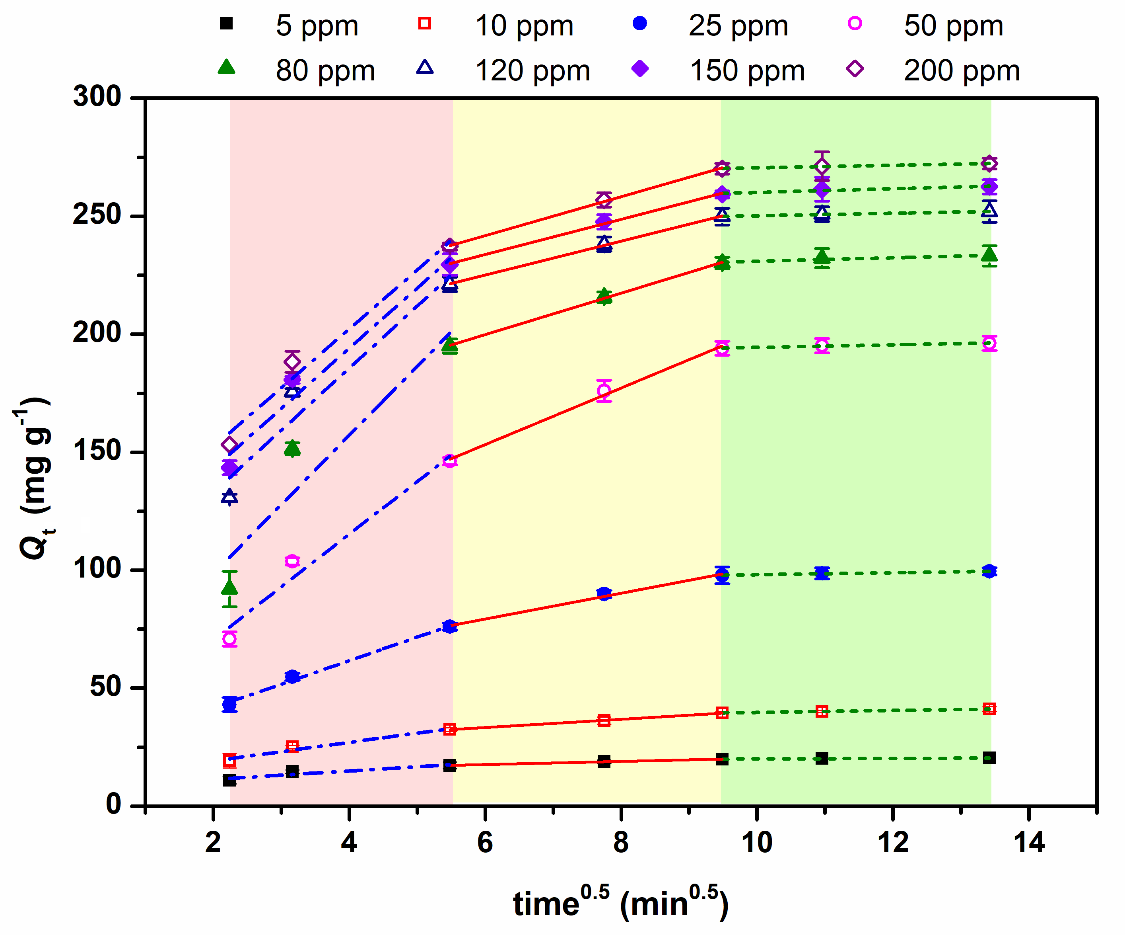


**Figure S4.** The linear fitting of the IPD kinetic model for different initial concentrations (pH: 4.0; $W$: 5.0 mg; $V$: 20 ml; $C_{i}$: 5-200 mg L^–1^; $T$: 293 K; $t$: 5-180 min).


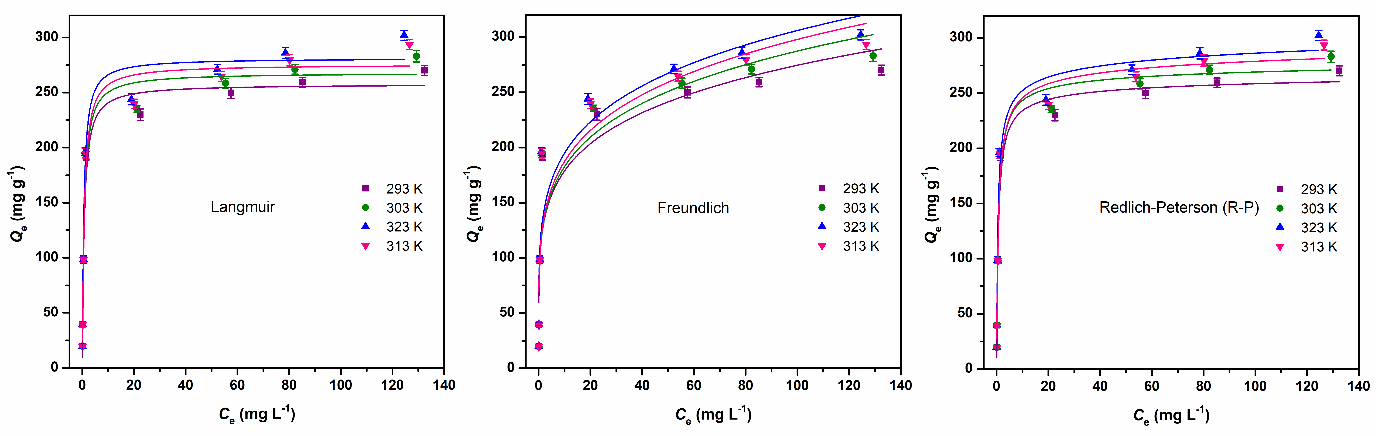


**Figure S5.** The experimental data and the nonlinear isotherms fitted to them at four different temperatures (pH: 4.0; $W$: 5.0 mg; $V$: 20 ml; $C_{i}$: 5-200 mg L^–1^; $T$: 293-313 K; $t$: 5-180 min)
